# The role of oxidative stress in the development of obesity and obesity-related metabolic disorders

**DOI:** 10.5937/jomb0-24652

**Published:** 2021-01-26

**Authors:** Emina Čolak, Dragana Pap

**Affiliations:** 1 Clinical Center of Serbia, Institute of Medical Biochemistry, Department for Scientific Research and Education, Belgrade; 2 Students Health Protection Institute, Department of Laboratory Diagnostics, Novi Sad; 3 University of Novi Sad, Faculty of Science

**Keywords:** adipokines, obesity, oxidative stress, metabolic disorders, inflammation, inflamacija, metabolički poremećaji, oksidativni stres, gojaznost, adipokini

## Abstract

Obesity is a serious medical condition, defined as excessive accumulation of fat. Abdominal fat is recognized as the major risk for obesity related diseases such as: hypertension, dyslipidemia, type 2 diabetes mellitus, coronary heart disease, stroke, non-alcoholic fatty liver disease etc. Fat accumulation is also related to pro-oxidant and pro-inflammatory states. Recently published articles suggest that oxidative stress may be a link between obesity and related complications. Adiposity leads to increased oxidative stress via several multiple biochemical processes such as superoxide generation through the action of NADPH oxidase, glyceraldehyde auto-oxidation, oxidative phosphorylation, protein kinase C (PKC) activation, and polyol and hexosamine pathways. On the other hand, oxidative stress plays a causative role in the development of obesity, by stimulating the deposition of adipose tissue, including preadipocyte proliferation, adipocyte differentiation and growth. Exercise-induced weight loss can improve the redox state by modulating both oxidative stress and antioxidant promoters, which reduce endothelial dysfunction and inflammation.

## Abstract (sr)

Gojaznost je ozbiljno zdravstveno stanje, definisano kao prekomerno nagomilavanje masti. Abdominalno masno tkivo predstavlja glavni rizik za razvoj bolesti povezane sa gojaznošcu kao što su: hipertenzija, dislipidemija, dijabetes melitus tipa 2, koronarna bolest srca, moždani udar, bezalkoholna masna jetra itd. Nagomilavanje masti je povezano i sa pro-oksidativnim i pro-inflamatornim stanjem. Nedavno objavljeni radovi ukazuju na to da oksidativni stres bi mogao predstavljati vezu između gojaznosti i komplikacija koje su vezane za gojaznost. Adipozitet vodi do povećanog oksidativnog stresa preko nekoliko biohemijskih procesa kao što su: generisanje superoksidnih radikala u reakcijama sa NADPH oksidazom, auto-oksidacija gliceraldehida, oksidativna fosforilacija, aktivacija protein kinaze C (PKC) i metabolički putevi poliola i heksozamina. Sa druge strane, oksidativni stres predstavlja ključni faktor u razvoju gojaznosti stimulacijom taloženja masnog tkiva uključujući proliferaciju preadipocita kao i rast i diferencijaciju adipocita. Gubitak telesne težine vežbanjem tj. fizičkom aktivnošću, može poboljšati redoks stanje organizma, modulacijom oksidativnog stresa i antioksidativnih promotera, koji smanjuju endotelnu disfunkciju i inflamaciju.

## Introduction

Obesity is a serious medical condition, defined as excessive accumulation of fat, related to impaired health and increased mortality [1]. The prevalence of obesity has recently become epidemic not only in developed countries but also in developing countries.

According to current assessment, it was indicated that more than 1 billion people worldwide were obese or overweight [2]. The classification of obesity is based on body mass index (BMI) defined as weight (kg) devided by square of height (m^2^). According to the values of BMI, there are three categories of values which indicate: normal range (BMI<25 kg/m^2^, overweight (BMI=25-30 kg/m^2^) and obesity (BMI>30 kg/m^2^) [1].

Abdominal fat is recognized as one of the major risk factor for obesity-related diseases such as: hypertension, dyslipidemia, metabolic syndrome, type 2 diabetes mellitus, coronary heart disease, stroke, non-alcoholic fatty liver disease etc [3] [4] [5] [6]. It is thought that fat accumulation also contributes, to pro-oxidant and pro-inflammatory states [2]. Excessive accumulation of fat is a consequens of positive energy balans resulting of several factors such as: increased intake of energy-rich food [7], decreased physical activity (sedentary lifestyle), nutritional and hormonal status [8], genetic, environmental, cultural and economic factors [9] [10].

## The role of oxidative stress in the pathogenesis of obesity

Oxidative stress could be a consequence but also a trigger for obesity. Increased intake of fats, carbohydrates, and saturated fatty acids, as well, especially trans-fatty acids, lead to increased oxidative stress via several biochemical processes such as the synthesis of superoxide anion via oxidative phosphorylation, glyceraldehyde auto-oxidation, protein kinase C (PKC) activation, and polyol and hexosamine pathways [11]. Oxidative stress also, plays a significant role in the development of obesity, by stimulating the deposition of adipose tissue, including preadipocyte proliferation, adipocyte differentiation and growth [12].

It has been documented that oxidative stress and inflammatory processes in obesity are strongly related [13]. Adipose tissue secretes proinflammatory cytokines such as: tumor-necrosis factor α (TNF-α), interleukin 1β (IL-1β) and IL-6, which induce the production of reactive oxygen species [14].

TNF-α influences the inflammatory response, apoptosis of adipose tissues and lipid metabolism, by increasing lipogenesis, insulin signalling and inducing oxidative stress and synthesis of reactive oxygen species. ROS trigger the release of inflammatory cytokines and pro-inflammatory transcription factors such as: nuclear factor kapa B (NF-κB), activator protein-1 (AP-1) which in turn increases the ROS production (*circulus vitiosus*) [15]. ROS induces the release of pro-inflammatory cytokines, adhesion molecules and growth factors including connective tissue growth factor, platelet-derived growth factor (PDGF), insulin-like growth factor-1 (IGF-1), and vascular cell adhesion molecule-1 (VCAM-1) [16], through redox-sensitive transcription factors, especially NF-κB and NADPH oxidase pathway [17].

TNF-α stimulates, also, the release of IL-6 [18]. The production of IL-6 could be enhanced by stimulation effect of IL-1β released from monocytes as a response to tissue damage, immunologic events, or infection [19]. IL-6 is synthesized by adipocytes, pancreatic β-cells, macrophages and monocytes. They regulate energy homeostasis and inflammation, by promoting the synthesis of pro-inflammatory cytokines [20]. It has been well documented that increased serum IL-6 concentrations are associated with the development of impaired glucose tolerance, diabetes mellitus, hypertension and obesity [21].

Excessive accumulation of fat in obese subjects is leading to a pathological increase of serum concentration of free fatty acids (FFA), which can impair glucose metabolism, stimulates the accumulation of energy substrates (glucose and fats) into hepatic, muscular and adipose tissue and initiates mitochondrial and peroxisomal oxidation [22]. These pathological conditions increase oxidative tissue damage, mitochondrial and DNA injury, depletion of adenosine triphosphate (ATP) and lipotoxicity [23]. Increased oxidative damage leds to higher production of cytokines, ROS synthesis and increased lipid peroxidation rate [24].

## The association of obesity and oxidative stress with metabolic syndrome

Obesity is considered the most important component of metabolic syndrome. According to the International Diabetes Federation criteria, metabolic syndrome existes, when three or more of the following features are present: obesity, hypertriglyceridemia, low HDL-cholesterol concentration, hyperglycemia, and hypertension [25]. A large number of adipocytokines (leptin, adiponectin, visfatin, PAI-1, resistin, TNF-α, and IL-6) are involved in the pathogenesis of metabolic syndrome (MS). Increased PAI-1 and TNF-α levels induce the development of thrombosis and insulin resistance [13]. Increased IL-6 levels are associated with BMI and insulin resistance. The last function is achieved through the impairment of hepatic signaling and affection of phosphorylation of insulin receptor substrate 1 (IRS-1), glucose transporter 4 (GLUT-4), and other transcription factors [26]. Leptin, also, affects the insulin sensitivity, inducing insulin resistance and lipid accumulation. Visfatin contributes to decreasing function of pancreatic β-cells [27]. Opposite to the effects of leptin and visfatin, adiponectin inhibits the activity of IL-6 and TNF-α (pro-inflammatory factors) and increases the production of IL-10 and IL-1 Ra (antiinflammatory factors) in adipocites and macrophages [28].

It is considered that oxidative stress has an important role in the development of metabolic syndrome by impairing the insulin secretion and glucose transport in adipose tissue and muscles [29]. Through the processes of lipid peroxidation, protein and DNA oxidation, locally produced reactive oxygen species, induce damage to cell structures including membranes, proteins and DNA. Excessive fatty acid accumulation and cytokines trigger systemic oxidative stress. On the other hand, increased body of evidence suggests that patients with metabolic syndrome show decreased systemic antioxidant defense system [30].

Oxidative stress is also, the cause of endothelial dysfunction, characterized by reduction of vasodilatator s bioavailability, especially nitric oxide (NO) and increase the endothelium-derived contractile factors [7]. The activation of endothelial cells is characterized by a pro-inflammatory, proliferative and pro-coagulant state, all favoring atherogenesis [30]. LDL-oxidation and increased expression of adhesion molecules in the endothelial layer, facilitate monocyte infiltration in the subendothelial space [31]. Oxidative stress increases vascular endothelial permeability and promotes leukocyte adhesion. Couillard et al. [32] found higher concentration of nitrotyrosine and superoxide ions associated with increased leptin concentration in the coronary endothelium of obese individuals.

Free fatty acids (FFA) are accumulated in nonadipose tissue in conditions of metabolic syndrome. During lipolysis, a higher concentration of FFA is delivered from mitochondria. Increased β-oxidation, impaired switching to carbohydrate substrate and decreased tricarboxylic acid (TCA) cycle activity, with products of incomplete oxidation can cause excessive production of superoxide anion through the mitochondrial electron transport chain [33]. Increased b-oxidation leads to increased mitochondrial NADH/NAD^+^ ratio, resulting in increased activation of protein kinase C (PKC), advanced glycosylation end products (AGE) and NF-κB [34]. Activated PKC, contributes to ROS production by increasing the activity of NADPH oxidase (NOX). Other effects include inhibition of nitric oxide synthase (eNOS) in endothelial cells, increased endothelial growth factor (VEGF) and decreased nitric oxide (NO) production in vascular smooth muscle cells. Activated PKC induces the activation of NF-κB and TGF-β, connecting in that way oxidative stress and inflammation [35]. The accumulation of AGE-s induces damage of cellular structure, through the activation of NOX, NF-κB, proinflammatory pathways, and cytokine synthesis [36].

## The impact of adiposity to insulin resistance

Insulin resistance represents a reduced ability of tissues to respond to insulin action. Insulin stimulates the storage of triglycerides in adipose tissue through a number of mechanisms: by promoting the differentiation of pre-adipocytes into adipocytes, by increasing the absorption of glucose and fatty acids, by increasing the lipogenesis in the mature adipocytes, and inhibiting lipolysis [37]. The effects of insulin are mediated through a complex pathway of signal transduction. The insulin signal transduction pathway is starting with binding of insulin to its receptor on the cell membrane, which leads to the activation of insulin receptor substrate protein (IRS). IRS is associated with the activation of phosphatidylinositol 3-kinase (PI3K)-Akt/protein kinase B (PKB), and pathway of the Ras-mitogen-activated protein kinase (MAPK). Activated IRS-1, induces the activation of PI3K by binding to its SH2 domain. PI3K stimulates the synthesis of phosphatidilinositol-(3,4,5)-tri phosphate, which acts as a »second messenger" in activation processes of several phosphatidilinositol-(3,4,5)-triphosphate-dependent serine/threonine kinases, including activated protein kinase B (PKB). These signaling processes, lead to the translocation of glucose transporter 4 (GLUT 4) in the plasma, resulting in increased glucose uptake into the adipocytes. The MAPK pathway is involved in the stimulation of growth and mitogenic effects of insulin. Insulin manifests anti-lipolytic effect in adipose tissue, via the activation of PI3K which stimulates phosphodiesterase-3, leading to increased hydrolysis of adenosine 3',5'-cyclic monophosphate in adipocytes. This process, in turn, limits the release of fatty acids from adipocytes [38].

There is evidence that some transcription factors, such as sterol regulatory element-binding protein-1c (SREBP1-c), can regulate the expression of genes responsible for lipogenesis, adipocyte differentiation, and fatty acid oxidation [38].

It has been suggested that some products of fatty acids metabolism, including ceramides, acylcoenzyme A, and diacyglycerol, can impair insulin signaling by promoting protein kinases (PKC, MAPK, JNK), and the inhibitor of NF-κB [39]. TNF-α also, stimulates ceramide accumulation through sphingomyelinase activation, while, ceramide in turn, mediates TNF-α-induced insulin resistance in adipocytes [40].

The adipocytokine regulation during insulin resistance is still unclear. It has been suggested, that one possible way was, via hexosamine biosynthetic pathway (HBP), which could result in insulin resistance and limitation the glucose concentration that can enter the cells, resulting in glucose toxicity [41]. The final product of the HBP is Uridine-diphosphate-N-acetylglucosamine (UDP-GlcNAc) which is, in fact the donor of sugar-group for the enzyme O-GlcNAc transferase (OGT) that adds the O-linked β-N-acetylglucosamine (O-GlcNAc) group to the serine and threonine residues of nucleo-cytosolic proteins [42]. It has been documented, that an increase in O-GlcNAc levels is sufficient to cause insulin resistance [43]. Furthermore, it has been also, documented, that the overexpression of OGT in the peripheral tissues of mice can cause insulin resistance and dysregulation of adipocytokine expression [41].

## Obesity, dyslipidemia and lipotoxicity

It is thought that lipotoxicity is associated with obesity and metabolic syndrome, and contributes significantly to organ dysfunction [44]. It involves the accumulation of non-esterified FFA and triglycerides into the cells. Abdominal adipose tissue synthesized increased levels of FFA [45]. Reduced adiponectin levels, leptin resistance, and the presence of other cytokines, originate from inflammatory cells and adipose tissue, reduce the FFA uptake into various tissues, reduce their oxidation, and stimulate its intracellular accumulation [46]. The intracellular accumulation of FFA and their metabolites leads to insulin resistance, which is accompanied by hyperinsulinemia and hyperglycemia. FFA exerts adverse effects on various organs, especially the cytotoxic effects on the liver, heart, pancreatic β cells, and endothelial cells. These changes lead to dys function of pancreatic β cells, cardiomyopathy, hepatic steatosis, and atherosclerosis [46]. These processes include the activation of protein kinase C (PKC), NF-κB, calpain-10 and oxidative stress, resulting in cellular necrosis, inflammation, and apoptosis [47]. FFA also stimulates the synthesis of VLDL and TG in the liver, which are metabolized into atherogenic LDL and oxidized LDL particles. These lipoproteins are lipotoxic. The transcription of many lipogenic genes is controlled by SREBP 8 (Sterol regulatory elementbinding protein 8), which is important regulator of cholesterol and fatty acid metabolism [48]. There are three SREBP isoforms: SREBP-1a, SREBP-1c, and SREBP-2. SREBP-1 activates genes involved in the synthesis of fatty acid, while, SREBP-2 activates genes that regulate the synthesis of cholesterol such as: genes for hydroxyl-methyl-glutaryl CoA (HMG-CoA) synthase and reductase [49].

Peroxisome proliferator-activated receptor (PPAR) is a nuclear hormone-activated receptor and transcription factor, having an important role in lipid metabolism, adipogenesis, and regulation of insulin sensitivity [50]. Three PPAR isoforms (PPAR-α, PPAR-ϒ, PPAR-δ) are expressed in various tissues, such as: adipose tissue, heart, liver, kidney and muscle [51]. It was documented that PPA receptors had specific roles in the body. For instance, PPAR-α, have an important role in promotion of FFA oxidation and insulin sensitivity [52]. The PPAR-δ and PPAR-ϒ induce insulin sensitivity and adipogenesis. They increase adiponectin activity and HDL concentration, reduce the synthesis of triglycerides and LDL particles, mediate cellular efflux of lipids, and modulate the activation of macrophages and foam cells in the process of atherosclerosis [51]. The agonists of PPAR-ϒ such as the thiazolidinediones could improve insulin sensitivity in the onset of type 2 diabetes [53]. From the above lines, we can understand that the SREBP and PPAR are very important factors in the metabolism of lipids and the onset of metabolic syndrome.

Accumulated lipids may cause renal and epithelial cell injury and may promote the progression of renal disease [54]. A significant overexpression of SREBP-1 and SREBP-2 protein, fatty acid synthase, acetyl CoA carboxylase, PAI-1, fibronectin and type IV collagen, in kidney, were found in obesity-prone C57/BL/6J mice, feeding with fat, together with increased renal accumulation of triglycerides and cholesterol in tubulointerstitial and glomerular cells [55]. A significant glomerulosclerosis and proteinuria was documented in these mice. Transgenic overexpression of SREBP-1a promoted lipid accumulation in glomerulus and tubules, inducing glomerulosclerosis, and tubulointerstitial injury [56]. The mechanisms that are associated with FFA lipotoxicity, and the release of non-polar lipids by proximal tubular cells (which attract macrophages and stimulate inflammation and apoptosis), are related to PPAR-ϒ FFAinduced apoptosis [57].

Lipid disorders related to obesity are characterized by increased levels of triglyceride and free fatty acids, reduced level of high density lipoproteins (HDL) and abnormal composition of low density lipoprotein (LDL). The uncontrolled release of FFA from adipose tissue during lipolysis, can cause an increased flux of fatty acids to the liver, and the synthesis of a very low-density lipoprotein particles (VLDL). The activity of lipoprotein lipase can be reduced in adipose tissue and muscles, as a result of increased concentrations of free fatty acids. The increased synthesis of VLDL may inhibit the lipolysis of chylomicrons, which leads to hypertriglyceridemia [58]. Hypertriglyceridemia triggers the exchange of triglycerides and cholesterol esters between intermediate VLDL and lipoproteins, which leads to a decreased HDL-cholesterol and reduction of triglyceride content in LDL. These triglycerides are further, hydrolyzed by the action of hepatic lipase (HL), which leads to formation of small, dense LDL particles [59]. It is well documented, that high concentrations of small, dense LDL cholesterol are highly associated with increased risk for cardiovascular disease [60]. Increased delivery of free fatty acids and increased triglyceride synthesis in the liver, exacerbates insulin resistance.

Sam et al. [61] found a positive association of dyslipidemia and visceral adipose tissue in type 2 diabetic patients. It was also, documented a positive relationship between visceral adipose tissue, large VLDL particles, small LDL and HDL particles. Enlarged visceral adipose tissue correlated positively with the activity of hepatic triglyceride lipase (HL) and increased cardiovascular risk. A lot of inflammatory molecules, synthesized by adipose tissues such as: IL-1, TNF-α, IL-6, adiponectin, serum amyloid A (SAA), and macrophages, could play an important role in the development of dyslipidemia [62]. Cancello and coworkers documented that there was a positive correlation between macrophage infiltration into visceral adipose tissue and serum triglyceride levels in obese patients, and negative correlation with plasma HDL-cholesterol concentration [63]. It has been suggested that CD68-a macrophage-specific marker positively correlated with serum free fatty acid concentration as well as with LDL-cholesterol levels and negatively correlated with HDL-cholesterol levels [64]. Furthermore, the size, composition and function of the HDLs may be modified by inflammation, which leads to a reduction of reverse cholesterol transport. Changes in apolipoproteins, cholesterol metabolism-related enzymes, and antioxidant capacity of adenosine triphosphate binding cassette A1-dependent efflux are also, posible. Some adipokines, such as IL-6 and TNF-α, can stimulate lipolysis and reduce the clearance of triglyceride-rich particles, by suppressing the LPL activity [65].

## The relation of adiposity and oxidative stress to non-alcoholic fatty liver disease (NAFLD)

Increased prevalence of nonalcoholic fatty liver disease (NAFLD) is highly associated with increased frequency of obesity. NAFLD is characterized by: increased accumulation of triglycerides in the liver (hepatic steatosis) [2], inflammation and subsequent fibrosis (nonalcoholic steatohepatitis (NASH) [66]. A lot of biochemical processes, including oxidative stress, mitochondrial dysfunction, increased expression of pro-inflammatory adipokines and cytokines, and subsequent lipid peroxidation, are responsible for the initiation of NAFLD [67]. The imbalance of lipid metabolism, and insulin resistance are considered to be the initial step for the development of NAFLD [68]. Hyperinsulinemia, as a consequence of insulin resistance, leads to hepatic steatosis, via increased lipogenesis, reduced fatty acid oxidation and increased efflux of free fatty acids that occur due to increased lipolysis in adipocytes, as well as reduced secretion of VLDL particles in the liver [69]. In steatosis, liver becomes more vulnerable to oxidative damage, adipokine/cytokine imbalance, apoptosis, mitochondrial dysfunction, pro-fibrogenic and proinflammatory mediators from damaged organelles of hepatic and Kupffer cells [38]. Excessive lipid accumulation in the liver occurs when the influx of lipids exceeds the ability of hepatic lipid clearance [70].

Enlarged adipose tissue could initiate the secretion of pro-inflammatory cytokines and adipokines and macrophage infiltration which are closely related to insulin resistance. The insulin inability of reducing lipolysis, leads to increased release of free fatty acids from adipose tissue and their influx directly into the liver. These changes are resulting in free fatty acid accumulation, reduced insulin clearance with an increase in circulating insulin levels [71]. The action of FFA could increase the production of glucose and triglyceride and exacerbates the insulin suppression of hepatic glucose output, via the membrane-bound TLR4, and promote inflammation [72]. Inflamed adipose tissue secretes pro-inflammatory and antiinflammatory factors, which are also associated with NAFLD [73].

It is suggested that, adiponectin could protect the liver from steatosis and inflammation, by increasing the ability of insulin to suppress glucose production and glucose output [74]. Adiponectin can inhibit lipogenesis by down-regulation of SREBP1-c and promotion of glucose utilization and fatty-acid oxidation in the liver by AMPK [75]. The anti-inflammatory properties of adiponectin might suppress the progression of hepatic steatosis to fibrosis [76]. According to some clinical studies, it has been suggested that adiponectin has a protective role in NAFLD. It has been documented that adiponectin levels were significantly lower in subjects with NAFLD compared to healthy controls [77]. According to some authors, reduced adiponectin levels may predict liver steatosis and increased liver enzyme levels in obese patients. Kaser at al. [78] showed that expression of adiponectin and its receptor (*AdipoR2*) were significantly reduced in patients with NASH compared with those with simple steatosis.

Leptin is also, an important regulator of NAFLD, by stimulating AMPK which is involved in activation of β-oxidation and glycolysis, and inhibition of lipogenesis [79]. Serin and his coworkers reported a negative correlation between serum leptin levels and hepatic injury in humans [80]. Poordad et al. [81] documented that serum leptin concentration positively correlated with serum ALT activity in patients with hepatic steatosis, which was independent of BMI or body fat mass. Čolak et al. [70] proved that obese students, aged 18-29 yrs. with higher BMI, WC (waist circumference), HC (hip circumference) and WHR (waist and hip ratio) values had higher activities of liver enzymes AST, ALT and gGT, and higher probability to develop NAFLD in the future. In animal models, the lack of leptin causes a reduction of liver damage, while an increase of its concentration causes hepatic fibrosis [82]. Leptin has the ability to increase the expression of pro-fibrogenic cytokine-transforming growth factor-β1) in Kupffer cells, activates hepatic cells and stimulates production of collagen, actin, and tissue inhibitor of metalloproteinase-1 [83]. Leptin has a potent mitogen activity on hepatic cells, inhibitis stellate cells apoptosis, and promotes the liver fibrosis processes [84].

Resistin, another adipokine, regulates lipid and glucose metabolism and could mediate the insulin resistance. It was documented that NAFLD patients had higher levels of circulating resistin [85]. TNF-α could induce both, the early stages of NAFLD and the development of more advanced stages of liver damage. Moreover, IL-6 and TNF-α, are involved in increased hepatic SREBP-1c expression and insulin resistance, via the increased expression of SOCS in the liver [86].

## Conclusion and future directions

The occurrence of obesity in children and adults is increasing rapidly in high-income, middle and lowincome countries. Obesity is a major risk factor for severe pathologies including metabolic syndrome, type 2 diabetes, NAFLD, hypertension, coronary heart disease, stroke, cancer etc, implying increased mortality and morbidity rates, as well as high health care costs.

A lot of research is now performing, in order to detect and identify molecular changes and genetic susceptibility of diseases related to obesity, in order to identify and determine the objectives of the strategy for preventing obesity [1]. According to the available measured data, obesity is closely associated with changes in the redox state, leading to subsequent development of different co-morbidities. Weight loss caused by physical activity in combination with carefully selected diet, it seems to be the most effective approach to minimize the oxidative stress and the risk of complications in obese patients [87].

## Conflict of interest statement

The authors state that they have no conflicts of interest regarding the publication of this article.

## List of abbreviations

TRL-4, Tool like receptor 4; NAFLD, nonalcoholic fatty liver disease; NASH – non-alcoholic steatohepatitis; SREBP, sterol regulatory element binding protein; AMPK – AMP activated protein kinase; Adipo R2, adiponectin receptor 2; BMI, body mass index; TNF-α, tumor necrosis factor alpha; SOCS, suppressor of cytokine signaling; NADPH, nicotinamidadenin dinucleotide phosphate; ROS, reactive oxygen species; FFA, free fatty acids; NOS, nitric oxyde synthase; NO, nitric oxyde; NOX, NADPH oxydase; MAPK, mitogen activated protein kinase; ERK, extracellular signal-regulated kinase; PKB, Protein kinase B; PKC, Protein kinase C; GLUT-4, glucose transporter 4; TCA, tricarboxylic acid; PPAR, peroxisome proliferator-activated receptors; SAA, serum amyloid A; CD68, cluster of differentiation 68; LPL, lipoprotein lipase; AKT/PKB, serin/threonin protein kinase B; AGE, advanced glycosylated end products; JNK-c-Jun N-terminal kinase; PI3K-phosphatidylinozitol 3-kinase; IRS-Insulin receptor supstrate; UDP-GlcNAc-Uridinediphosphate-N-acetylglucosamine; O'-GlcNAc-O-linked b N-acetylglucosamine; OGT-O-linked β N-acetyl glucosamine transferase.
